# Changes in salivary microbiota increase volatile sulfur compounds production in healthy male subjects with academic-related chronic stress

**DOI:** 10.1371/journal.pone.0173686

**Published:** 2017-03-20

**Authors:** Bruno Dias Nani, Patricia Oliveira de Lima, Fernanda Klein Marcondes, Francisco Carlos Groppo, Gustavo Sattolo Rolim, Antonio Bento Alves de Moraes, Karina Cogo-Müller, Michelle Franz-Montan

**Affiliations:** 1 Department of Physiological Sciences, Piracicaba Dental School, University of Campinas—UNICAMP, Piracicaba, SP, Brazil; 2 Department of Basic Health/Applied Psychology, Federal University of Juiz de Fora (UFJF), Governador Valadares, MG, Brazil; National Institute of Dental and Craniofacial Research, UNITED STATES

## Abstract

**Objective:**

To investigate the associations among salivary bacteria, oral emanations of volatile sulfur compounds, and academic-related chronic stress in healthy male subjects.

**Materials and methods:**

Seventy-eight healthy male undergraduate dental students were classified as stressed or not by evaluation of burnout, a syndrome attributed to academic-related chronic stress. This evaluation was carried out using the Maslach Burnout Inventory—Student Survey questionnaire. Oral emanations of hydrogen sulfide, methyl mercaptan, and dimethyl sulfide were measured using an Oral Chroma™ portable gas chromatograph. The amounts in saliva of total bacteria and seven bacteria associated with halitosis were quantified by qPCR. The *in vitro* production of H_2_S by *S*. *moorei* and/or *F*. *nucleatum* was also measured with the Oral Chroma™ instrument.

**Results:**

The stressed students group showed increased oral emanations of hydrogen sulfide and dimethyl sulfide, together with higher salivary *Solobacterium moorei* levels (p < 0.05, Mann Whitney test). There were moderate positive correlations between the following pairs of variables: *Fusobacterium nucleatum* and *S*. *moorei*; *F*. *nucleatum* and hydrogen sulfide; *Tannerella forsythia* and *F*. *nucleatum*; *T*. *forsythia* and *S*. *moorei*. These correlations only occurred for the stressed group (p < 0.05, Spearman correlation). The *in vitro* experiment demonstrated that *S*. *moorei* increased H_2_S production by *F*. *nucleatum* (p < 0.05, ANOVA and Tukey’s test).

**Conclusion:**

The increased amount of *S*. *moorei* in saliva, and its coexistence with *F*. *nucleatum* and *T*. *forsythia*, seemed to be responsible for increased oral hydrogen sulfide in the healthy male stressed subjects.

## Introduction

Halitosis can be a physiological and/or a pathological condition [1]. In both cases, it is characterized by malodorous gases released from the mouth and/or the nose, and can originate in the oral cavity, the respiratory tract, the stomach, systemically, or due to various combinations of these sources [2].

Oral malodor, or “morning breath”, is common on awakening, due to the physiological reduction in salivary flow during sleep and the lack of physiological oral cleansing promoted by the facial muscles, resulting in increased microbial metabolic activity [3]. This condition is transient, disappearing following normal oral hygiene after awakening. However, pathological halitosis is more intense, persistent, and offensive, requiring treatment to eliminate its cause, and can be due to extra- or intra-oral factors [4]. Physiological halitosis can be persistent, but may not be offensive to others, while the pathological condition is offensive and can continue while its source exists [2].

It is known that anaerobic bacteria can degrade sulfur-containing amino acids, producing the volatile sulfur compounds (VSCs) that are the major causative agents of oral malodor [5]. The main VSCs involved are hydrogen sulfide (H_2_S), methyl mercaptan (CH_3_SH), and dimethyl sulfide ((CH_3_)_2_S) [6].

Gram-negative anaerobic periodontopathogenic bacteria such as *Porphyromonas gingivalis*, *Tannerella forsythia*, *Treponema denticola*, and *Fusobacterium nucleatum* are some of the most important VSCs producers [7, 8]. Furthermore, it has been reported that bacteria of the *Actinomyces* and *Veillonella* genera, especially the commensal bacteria *Actinomyces odontolyticus* (Gram-positive) *and Veillonella dispar* (Gram-negative), are associated with malodor formation [9]. Higher levels of *Solobacterium moorei*, a Gram-positive bacterium, have also been found in the tongue coatings and saliva of patients with halitosis [10–12].

It has been shown that anxiogenic experimental situations [13, 14] and academic examinations [15] can lead to increased oral H_2_S production in healthy subjects, demonstrating a positive influence of anxiety and psychological stress on oral VSCs emanation. In these cases, the VSCs production may not necessarily be considered halitosis, but when associated with other factors, such as poor oral hygiene or periodontal diseases, increased production of these compounds may cause halitosis.

Although the relationship between psychological stress and VSCs production has been reported [13–15], the mechanisms involved remain unclear. Some studies have demonstrated that stress-related substances can affect the growth of several periodontopathogens [16,17], upregulate the expression of virulence and oxidative stress genes in *P*. *gingivalis* [18], and increase H_2_S and CH_3_SH production by *F*. *nucleatum* [19].

Therefore, we hypothesized that the alteration of saliva composition by academic-related chronic stress could create a favorable environment for bacterial VSCs production, resulting in increases of VSCs in these subjects. The aim of the present study was to evaluate the relationships among the quantity of bacteria in saliva, oral emanation of VSCs, and academic-related chronic stress in healthy male subjects.

## Materials and methods

### Clinical study

#### Experimental design

**Fig 1:**
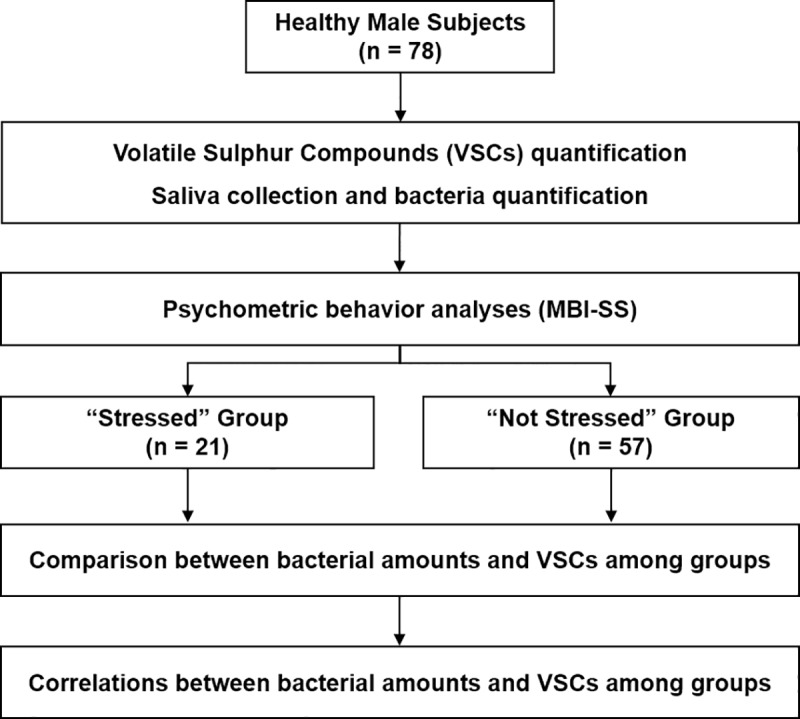
Flowchart of experimental design.

In order to avoid the influence of the menstrual cycle, only male volunteers were invited to participate in the present study. Seventy-eight healthy male subjects provided saliva for quantification of bacterial species producing VSCs. The VSCs (H_2_S, CH_3_SH, and (CH_3_)_2_S) were also quantified in the oral breath. The volunteers were classified as either “Stressed” (n = 21) or “Not stressed” (n = 57) using psychometric behavior analysis (Maslach Burnout Inventory Student Survey questionnaire; MBI-SS), a validated tool for determination of a state of academic-related chronic stress. Comparisons between oral VSC and salivary bacterial levels, correlations among bacterial species and between bacterial species and oral VSC emanation were made (Fig 1).

#### Participants

Selection was made of seventy-eight male undergraduate students at Piracicaba Dental School, aged between 18 and 24 years and in good oral and systemic health. Clinical evaluation was made of the tongue coating (measured using an index described elsewhere [19]), caries, third molar eruption, dental plaque and gingival indexes, and probing depth. Exclusion criteria were a tongue coating index equal to or greater than one, plaque and bleeding index values higher than 10%, presence of periodontal pockets, defective restorations, prostheses, caries, third molars in eruption, smoking habit, and systemic disease. These criteria were implemented to ensure that all the volunteers had good oral and systemic health and that chronic academic stress was the distinguishing factor between the groups. This study was approved by the Research Ethics Committee of Piracicaba Dental School, University of Campinas, Piracicaba, Brazil (protocol numbers #108/2007 and #147/2014), and the volunteers provided written informed consents.

#### Clinical data (collection of saliva and VSCs quantification)

Clinical data were obtained between 7:00 and 8:00 a.m. in the second semester of the year, on three different days (0, 7, and 15) within a 15-day period. These dates were scheduled after stressful events such as seminars, exams, or any kind of academic work that might cause worry in the students concerning their approval in the disciplines. These precautions were taken in order to ensure that the stress conditions were strictly chronic, since acute stressful circumstances (such as biochemistry exams or an anxiogenic experimental situation) have been previously reported as being capable of increasing VSCs production [13, 15]. The averages of the three collections were used, since this short period was not sufficient to observe significant changes in oral mature biofilms in young adults [20].

The subjects were instructed to refrain from using any oral rinse or breath freshener for a week, avoid eating spicy foods or those containing onion or garlic for 24 h, and not to practice their oral hygiene routines, use scented cosmetics or after-shave lotions, or eat and drink (including water) for 8 h before the experiment [13].

The unstimulated saliva collection was carried out as recommended elsewhere [13, 16]. The subjects were instructed to avoid swallowing for 5 min. Subsequently, the total volume of saliva was placed in a plastic tube kept on ice. Immediately after collection, the saliva was homogenized using a vortex mixer and stored in a freezer at -70°C until analysis.

Oral breath samples (1 mL of mouth air) were obtained with disposable plastic syringes, after 1 min of keeping the mouth closed. The VSCs measurements were performed by injecting 0.5 mL of the sample into an Oral Chroma™ (Ability, Osaka, Japan) portable gas chromatograph, which separated and quantified (in ppb) H_2_S, CH_3_SH, and (CH_3_)_2_S.

#### Salivary bacteria quantification

Quantitative microbial analyses were performed with saliva samples collected as described above from each volunteer on the three collection days. Quantitative real-time polymerase chain reactions (qPCR) were carried out in order to obtain total bacteria counts and quantify the main oral bacterial species that produced VSCs. A 520 μL volume of saliva was centrifuged at 13,523 *g* for 10 min at 4°C. The supernatant was discarded and the pellet was used for DNA extraction using a genomic DNA detection kit (PureLink^TM^ Genomic DNA Mini Kit, Invitrogen, Carlsbad, CA, USA). The protocol was performed according to the manufacturer’s instructions, with minor modification of the lysis time (Proteinase K and Purelink^TM^ Genomic Lysis/Binding Buffer step), which was changed to 2 h. The final elution was made using 25 μL of elution buffer. Following extraction, the DNA concentration was determined using a small volume PICOPET01 spectrophotometer (Picodrop Ltd., Alpha Biotech Ltd., Killearn, Glasgow, Scotland).

All species-specific primers for *A*. *odontolyticus*, *T*. *forsythia*, *T*. *denticola*, *P*. *gingivalis*, and total bacterial counts targeted the *16SrRNA* gene [21–25]. Since the *Fusobacterium* and *Veillonella* genera have high levels of genotypic similarity among their species, primers for *F*. *nucleatum* and *V*. *dispar* targeted the *rpoB* gene, which is a species-specific gene [26, 27]. The *S*. *moorei* primer targeting the *16SrRNA* gene was designed using the Primer 3 software [28]. All primers were tested for specificity using the NCBI BLAST database [29]. Table 1 shows the sequences of primers used in this study.

**Table 1:** Species-specific primers used in the real-time PCR.

Bacteria		Primer sequences (5' → 3')	References
*A*. *odontolyticus*	F	CTTTGGGATAACGCCGGGAAAC	[21]
	R	CTACCCGTCAAAGCCTTGGT	
*F*. *nucleatum*	F	ACCTAAGGGAGAAACAGAACCA	[26]
	R	CCTGCCTTTAATTCATCTCCAT	
*P*. *gingivalis*	F	ACCTTACCCGGGATTGAAATG	[24]
	R	CAACCATGCAGCACCTACATAGAA	
*S*. *moorei*	F	CTCAACCCAATCCAGCCACT	Designed in this study
	R	TATTGGCTCCCCACGGTTTC	
*T*. *forsythia*	F	AGCGATGGTAGCAATACCTGTC	[22]
	R	TTCGCCGGGTTATCCCTC	
*T*. *denticola*	F	CCGAATGTGCTCATTTACATAAAGGT	[23]
	R	GATACCCATCGTTGCCTTGGT	
Total bacterial counts	F	TGGAGCATGTGGTTTAATTCGA	[25]
	R	TGCGGGACTTAACCCAACA	
*V*. *dispar*	F	AACGCGTTGAAATTCGTCATGAAC	[27]
	R	GTGTAACAAGGGAGTACGGACC	

The qPCR reaction was carried out in a total volume of 10 μL, containing 5 μL of SYBR^®^ Select Master Mix (Thermo Fisher Scientific, Waltham, USA), 2 μL of DNA template, and 1 μL of primer pair solution (300 nM/reaction). For each run, DEPC-treated water (Thermo Fisher Scientific, Waltham, USA) was used as the negative control and melting peaks were used to determine the speciFIcity of the PCR. Amplification of the extracted DNA template was performed using a real-time PCR system (Step One Plus^®^, Thermo Fisher Scientific, Waltham, USA), with an initial incubation of 2 min at 50°C and 2 min at 95°C, followed by 40 cycles of 15 s at 95°C and 30 s at 60°C.

The absolute quantification of the target bacteria was performed by comparing the Ct values of the saliva samples with Ct values from a standard curve (10^2^–10^8^ CFU/mL) constructed using pure cultures of the bacterial species studied [30]. Calculations were carried out to obtain the number of bacteria per mL of saliva (cells/mL).

#### Psychometric behavior analyses (MBI-SS)

Following data collection, the volunteers were classified into two groups: G1 - “Stressed” or G2 - “Not stressed”. Stress was evaluated by quantification of burnout syndrome, a state associated with long-term work-related mental exhaustion, accompanied by diminished interest and disengagement [31], which is directly related to perceived stress [32]. The volunteers were invited to answer the MBI-SS questionnaire (S1 and S2 Figs), which is used to detect burnout syndrome in undergraduate dental students [31, 32]. This syndrome is triggered by academic-related chronic stress and it is characterized by a high degree of emotional exhaustion, cynicism, and low academic efficacy [31].

The questionnaire consists of 15 questions divided into three categories: exhaustion (5 items), cynicism (4 items), and efficacy (6 items). All questions are scored on a 7-point frequency rating scale ranging from 0 (never) to 6 (every day). This evaluation has a three-dimensional burnout concept, with high scores for emotional exhaustion and cynicism and low scores for academic efficacy indicating a high degree of burnout. Classification of the volunteers was performed according to the sum of scores for each category (range 0–90 points). The volunteers were considered as “stressed” when the scores sum was above the third quartile, and as “not stressed” when the scores sum was below the third quartile [31].

#### Clinical statistical analysis

The sample size was calculated *a priori*, considering a difference of 30% between the means of the two groups, and a standard deviation of 20% of the mean. In this scenario, a sample size of 12 volunteers in both groups provides 99% of power, considering 5% for alpha. The study included 78 volunteers (21 in a stressed condition and 57 control subjects), representing almost all the male undergraduate students enrolled at the faculty during the study period. The data distribution was tested using the Shapiro-Wilk test. Non-normally distributed data (bacterial quantification and clinical VSCs determination) were analyzed using the Mann-Whitney U test. Spearman correlation coefficients (rS) were calculated for VSCs production and bacterial quantification, with rS values of 0.2–0.4, 0.4–0.8, and >0.8 considered to indicate weak, moderate, and strong correlations, respectively [33]. For bacterial quantification, the data were expressed as percentages representing the proportion of each species relative to the total quantification. The significance level was set at 5%, and all analyses were performed using GraphPad Prism v. 6.0 for Windows statistical software (GraphPad Software Inc., Los Angeles, USA).

### *In vitro* study

#### Bacteria and culture conditions

*S*. *moorei* (DSM 22971) was kept on Bacto™ Tryptic Soy Agar (TSA; Difco, Le Pont de Claix, France) supplemented with sheep blood (7% v/v). *F*. *nucleatum* (NCTC 11326) was kept on TSA supplemented with 0.2% Bacto™ Yeast Extract (YE; Difco, Le Pont de Claix, France), 5 μg/mL of hemin (HE; Sigma, Poole, UK), 1 μg/mL of menadione (ME; Sigma, Poole, UK), and 5% (v/v) sheep blood. The cultures were grown under anaerobic conditions (10% CO_2_, 10% H_2_, 80% N_2_) in an anaerobic chamber (MiniMacs Anaerobic Workstation, Don Whitley Scientific, Shipley, UK) at 37°C. For the VSCs assays, *S*. *moorei* was cultured in Bacto™ Tryptic Soy Broth (TSB; Difco, Le Pont de Claix, France), while *F*. *nucleatum* was cultured in TSB supplemented with yeast extract, hemin, and menadione.

#### *In vitro* VSCs assay

An *in vitro* VSCs assay was carried out to test the hypothesis that *S*. *moorei* and *F*. *nucleatum* cultures in combination could produce higher amounts of VSCs, compared to the individual cultures. This assay was performed as described previously [7]. Briefly, *S*. *moorei* and *F*. *nucleatum* were cultured in broth until reaching the logarithmic phase (0.5 < optical density < 1.0) and were then centrifuged at 2876 *g* for 5 min. The pellets were washed and the bacterial inoculum was prepared in phosphate buffer saline (PBS; pH 7.7) at an optical density of 0.1 at 660 nm.

The following suspensions were kept in 20 mL headspace vials and incubated at 37°C for 1 h: **1) *S*. *moorei***—100 μL of *S*. *moorei* inoculum + 885 μL of PBS + 15 μL of 33 mM L-cysteine (Sigma, Poole, UK); **2) *F*. *nucleatum*—**100 μL of *F*. *nucleatum* inoculum + 885 μL of PBS + 15 μL of 33 mM L-cysteine; **3) *S*. *moorei* + *F*. *nucleatum*—**100 μL of *S*. *moorei* inoculum + 100 μL of *F*. *nucleatum* inoculum + 785 μL of PBS + 15 μL of 33 mM L-cysteine.

After incubation, the reaction was stopped by the addition of 500 μL of 3 M phosphoric acid during 10 min. A 1 mL volume of the headspace gas was sampled and the H_2_S production was measured using the Oral Chroma™ system, as described in Section 2.1.3. Under these experimental conditions, CH_3_SH and (CH_3_)_2_S were not detectable. The experiment was carried out using nine replicates.

#### *In vitro* statistical analyses

Group comparisons were performed by ANOVA followed by Tukey's multiple comparisons test. The significance level was set at 5% and the analyses were performed using GraphPad v. 4.0 for Windows statistical software (GraphPad Software Inc., Los Angeles, USA).

## Results

**Fig 2:**
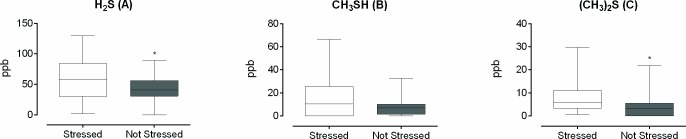
**Box plots of (A) H_2_S, (B) CH_3_SH, and (C) (CH_3_)_2_S produced by the volunteers in the “Stressed” and “Not stressed” groups.** Box plot explanation: upper horizontal line of box, maximum; lower horizontal line of box, minimum; horizontal bar within box, median; upper horizontal bar outside box, 75^th^ percentile; lower horizontal bar outside box, 25^th^ percentile. Mann-Whitney test, * p < 0.05.

The subjects were allocated to the “Stressed” and “Not stressed” groups based on the MBI-SS results (clinical data are available as supporting information–S1 File). The production of H_2_S, CH_3_SH, and (CH_3_)_2_S by volunteers in the two groups is shown in Fig 2.

The “Stressed” group presented higher levels of H_2_S (p = 0.03) and (CH_3_)_2_S (p = 0.004), compared to the “Not stressed” group. There was no significant difference between the groups for CH_3_SH production.

**Fig 3:**
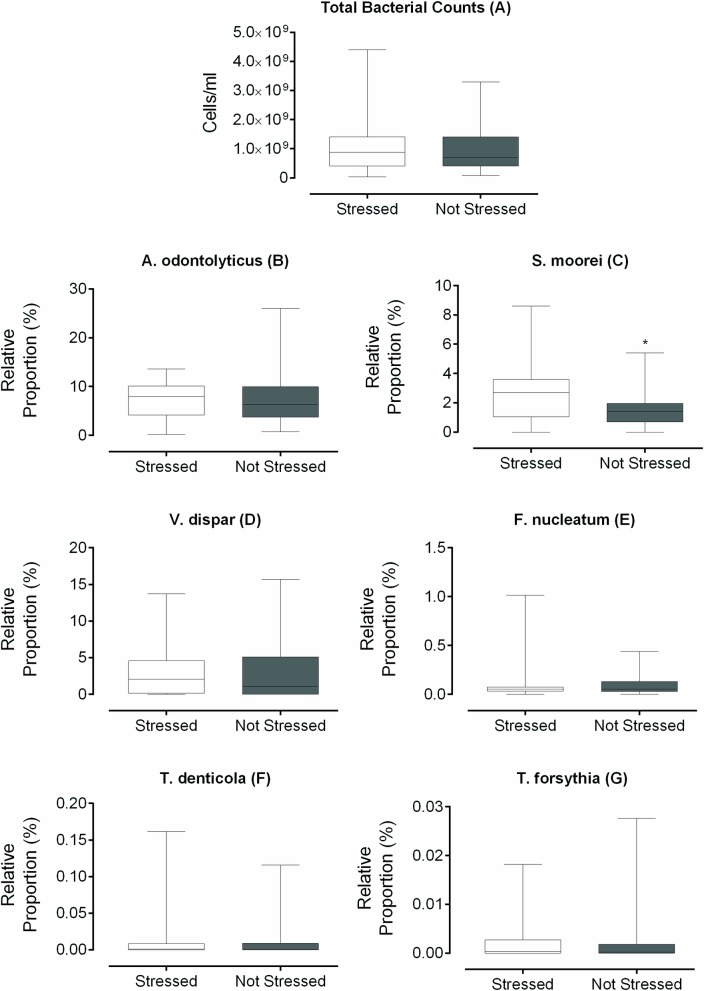
**Box plots of (A) total bacterial counts, and the relative proportions (percentage of the total bacteria) of (B) *A*. *odontolyticus*, (C) *S*. *moorei*, (D) *V*. *dispar*, (E) *F*. *nucleatum*, (F) *T*. *denticola*, and (G) *T*. *forsythia* in the saliva of volunteers from the “Stressed” and “Not stressed” groups.** Box plot explanation: upper horizontal line of box, maximum; lower horizontal line of box, minimum; horizontal bar within box, median; upper horizontal bar outside box, 75^th^ percentile; lower horizontal bar outside box, 25^th^ percentile. Mann-Whitney test, * p < 0.05.

The total bacterial counts and the relative proportions of bacterial species producing VSCs, including *A*. *odontolyticus*, *F*. *nucleatum*, *S*. *moorei*, *T*. *denticola*, *T*. *forsythia*, and *V*. *dispar*, in saliva samples of volunteers from the “Stressed” and “Not stressed” groups, are shown in Fig 3.

There was a statistically significant difference between the groups for salivary *S*. *moorei* (p = 0.01). For both groups, the saliva samples were negative for *P*. *gingivalis*.

Table 2 shows the relationship between the VSCs values and the amounts of bacterial in saliva (Spearman correlation test).

**Table 2:** Correlation coefficients (rS) between H_2_S, CH_3_SH, and (CH_3_)_2_S and the relative proportions of *A*. *odontolyticus*, *F*. *nucleatum*, *S*. *moorei*, *T*. *denticola*, *T*. *forsythia*, and *V*. *dispar* in the saliva of subjects from the “Stressed” and “Not stressed” groups.

	Stressed			Not Stressed		
	H_2_S	CH_3_SH	(CH_3_)_2_S	H_2_S	CH_3_SH	(CH_3_)_2_S
*A*. *odontolyticus*	-0.18	0.00	0.40	0.15	0.15	0.05
*F*. *nucleatum*	0.51*	0.53*	0.29	-0.01	-0.09	-0.02
*S*. *moorei*	0.13	0.51*	0.42	0.25	0.25	0.16
*T*. *denticola*	-0.22	-0.05	0.18	-0.13	-0.03	-0.01
*T*. *forsythia*	0.38	0.24	0.23	-0.01	-0.19	0.06
*V*. *dispar*	0.00	0.01	-0.01	-0.07	0.08	-0.29

Spearman´s correlation test,

* p < 0.05.

There were moderate positive correlations between *F*. *nucleatum* and H_2_S (p = 0.006), *F*. *nucleatum* and CH_3_SH (p = 0.01), and *S*. *moorei* and CH_3_SH (p = 0.008), but only in the “Stressed” group (Table 2).

The data obtained (Fig 3 and Table 2) suggested that *S*. *moorei* and *F*. *nucleatum* could have been responsible for the increased VSCs production in the “Stressed” group (Fig 2). Therefore, a Spearman correlation test was performed in order to determine the relationships between the relative proportions of *S*. *moorei* or *F*. *nucleatum* and the relative proportions of other bacterial species, for the “Stressed” and “Not stressed” groups. The results are shown in Table 3.

**Table 3:** Correlation coefficients (rS) between the relative proportions of *S*. *moorei* or *F*. *nucleatum* and the relative proportions of *A*. *odontolyticus* (*Ao*), *T*. *denticola* (*Td*), *T*. *forsythia* (*Tf*), and *V*. *dispar* (*Vd*), for the “Stressed” and “Not stressed” groups.

	Stressed					Not stressed				
	*Ao*	*Fn*	*Td*	*Tf*	*Vd*	*Ao*	*Fn*	*Td*	*Tf*	*Vd*
*S*. *moorei*	0.46 *	0.59 *	0.36	0.53 *	0.16	0.61 *	0.08	0.18	0.09	0.35
*F*. *nucleatum*	0.20	-	0.09	0.55 *	0.31	0.10	-	0.11	0.25	-0.17

Spearman´s correlation test

* p < 0.01.

There were moderate positive correlations between *A*. *odontolyticus* and *S*. *moorei* for the subjects of the “Stressed” (p = 0.02) and “Not stressed” (p < 0.001) groups. On the other hand, moderate positive correlations between *F*. *nucleatum and S*. *moorei* (p = 0.02), *T*. *forsythia* and *S*. *moorei* (p = 0.02), and *T*. *forsythia* and *F*. *nucleatum* (p = 0.04) were only found for the “Stressed” group.

**Fig 4:**
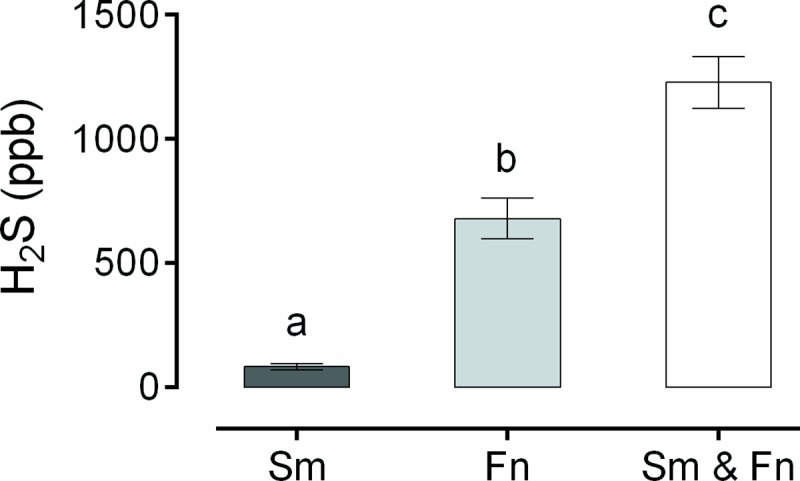
Means and standard deviations for the *in vitro* production of H_2_S (ppb) by *S*. *moorei* (Sm), *F*. *nucleatum* (Fn) and the H_2_S production by Sm and Fn when they were grown together (Sm & Fn). Different letters indicate statistically significant differences between groups (ANOVA and Tukey’s test, p < 0.05).

Based on these clinical findings, an *in vitro* experiment was carried out to demonstrate that *S*. *moorei* and *F*. *nucleatum* could interact and produce greater amounts of H_2_S, compared to production by the individual bacteria. The results are shown in Fig 4.

The H_2_S production obtained for the co-culture of *S*. *moorei* and *F*. *nucleatum* was significantly higher than the H_2_S production values for *S*. *moorei* or *F*. *nucleatum* grown individually (ANOVA and Tukey’s test, p < 0.0001).

## Discussion

Halitosis is characterized by an emanation of malodorous compounds from the oral cavity [1]. The main gases involved are H_2_S, CH_3_SH, and (CH_3_)_2_S, which are produced by anaerobic microorganisms during the degradation of sulfur-containing proteins [5]. It has been shown that psychological stress and anxiety are able to increase the oral VSCs emanation [13–15], but the mechanisms involved are not still understood.

In the present study, we found increased oral production of H_2_S and (CH_3_)_2_S, together with a higher proportion of salivary *S*. *moorei*, in the case of the stressed subjects (Figs 2 and 3). In addition, correlations were observed between oral VSCs and *F*. *nucleatum* and *S*. *moorei* (Table 2), and among *S*. *moorei*, *F*. *nucleatum*, and *T*. *forsythia* (Table 3). We believe that these data indicate that the increase in H_2_S production in stressed subjects can be attributed to the coexistence of these bacteria in the oral cavity, especially considering *S*. *moorei* and *F*. *nucleatum*, since these bacteria produced higher amounts of H_2_S when cultured together, as demonstrated *in vitro* (Fig 4).

Oral VSCs emanations can be affected by many factors, such as periodontal diseases [34], presence of tongue coating [35], menstrual cycle and gender [15, 16], systemic diseases [35], and drinking [36] and eating habits [37]. In the present study, the effects of other factors, apart from stress, that could influence VSCs production were controlled, since the volunteers were all fasted non-alcoholic and non-smoker men with good oral and systemic health, as assessed during clinical examination. Therefore, the observed effects on VSCs production could not be explained by negligent oral hygiene.

Moreover, the MBI-SS questionnaires showed that 21 subjects had burnout syndrome. Since burnout syndrome is directly related to the perceived chronic stress [32], we assumed that chronic stress was the distinguishing factor between the groups in this study, with the “Stressed” group presenting higher amounts of H_2_S in the oral cavity. It is important to note that VSCs levels could be even higher in patients with oral disorders facing stressful conditions.

In previous work by our group, it was found that anxiogenic experimental situations [13, 14] and academic exams [15] resulted in increased VSCs production in healthy undergraduate students. Other studies have also reported that academic stress impaired oral health, with academic exams being implicated in plaque accumulation and gingival inflammation [34, 38].

However, none of these studies explained how these oral alterations occur. Here, we demonstrated that subjects with academic-related chronic stress presented higher proportions of *S*. *moorei* in the saliva and greater oral VSCs production. This finding is in agreement with the literature, since this anaerobic bacterium is related to halitosis, due to its higher prevalence in the tongue coating of patients with this disease [10, 39].

Saliva bacterial composition reflects the overall condition of the oral cavity [40], and a previous study also reported significant correlations between salivary *S*. *moorei* and VSCs production [12]. Furthermore, our results show that an increased relative proportion of *S*. *moorei* in saliva could be associated with greater VSCs production in stressed healthy undergraduate students.

It was reported elsewhere that *S*. *moorei* seemed to be a low VSCs producer, compared to other oral microorganisms, and was unable to produce CH_3_SH [41]. Here, we found a moderate positive correlation between *S*. *moorei* and CH_3_SH in stressed subjects, which suggests that the presence of a stress condition might be important for it. We also found that *S*. *moorei* did not produce CH_3_SH *in vitro*, even when exposed to different culture conditions (data not shown). Therefore, future studies are necessary to understand the possible correlation of *S*. *moorei* with CH_3_SH.

The relative proportion of *F*. *nucleatum* showed no significant difference between the “Stressed” and “Not stressed” groups, but presented moderate positive correlations with H_2_S and CH_3_SH levels, only in stressed subjects. These results are in accordance with the literature, since *F*. *nucleatum* has a moderate ability to produce H_2_S, but is one of the leading producers of CH_3_SH [7, 41]. Therefore, our findings indicate that *F*. *nucleatum* may have been responsible for higher levels of these gases in the “Stressed” group, with increased production possibly being due to a direct effect of stress in stimulation of VSCs production by *F*. *nucleatum*.

Another mechanism that could explain the increase in VSCs production by *F*. *nucleatum* is the ability of *S*. *moorei* to interact with other bacteria, including species of *Fusobacterium*, as suggested by other authors since these two bacteria were found to cause endodontic infection [42], wound infection [43], septic pulmonary embolization [44], and periodontitis [45].

In agreement with this hypothesis, *S*. *moorei* was found in higher amounts in the “Stressed” group and presented a moderate positive correlation with the presence of *F*. *nucleatum*. Moreover, *S*. *moorei* was correlated with increased production of CH_3_SH in the “Stressed” group, even though it is not able to produce this gas.

We believe that these bacteria may be responsible for increased VSCs production in stressed subjects, as *S*. *moorei* and *F*. *nucleatum* were correlated with each other and with VSCs production in subjects with stress. Based on these findings, we performed an *in vitro* experiment showing that the co-culture of *S*. *moorei* and *F*. *nucleatum* resulted in H_2_S levels that were 1.6-fold higher than the sum of the H_2_S production by these bacteria cultured individually.

Previously, it was shown that although Gram-positive microorganisms are weak producers of VSCs, they play an important role in malodor formation, because they are responsible for the first enzymatic step (deglycosylation) required for the subsequent degradation of proteins by Gram-negative species [46]. In light of this information, we suggest that *S*. *moorei* and *F*. *nucleatum* may interact, participating in different steps of VSCs production, with *S*. *moorei* (Gram-positive) being responsible for deglycosylation, and *F*. *nucleatum* and *T*. *forsythia* (Gram-negative) acting in subsequent protein degradation, resulting in raised VSCs levels in stressed subjects. However, there is little information in the literature concerning biochemical studies of bacterial communities and VSCs production, and further work is required to understand these interactions.

The relationship between psychological stress and the development of infectious diseases has been studied. Recently, the concept of “microbial endocrinology” was introduced, with it being shown that microorganisms can interact with stress-related substances and react by altering their metabolism, virulence, and growth profile [47]. Adrenaline and noradrenaline have been shown to affect the growth of periodontal microorganisms [17]. Moreover, cortisol, noradrenaline, and adrenaline were able to reduce the growth of *F*. *nucleatum* and *Porphyromonas endodontalis*, while increasing the production of H_2_S by these bacteria [19]. These studies corroborate our findings, showing that VSCs levels could be altered even when bacterial loads are not altered. In the present study, *F*. *nucleatum* levels were not altered, but this bacterium showed correlations with the levels of H_2_S and CH_3_SH in stressed subjects.

The concentrations of stress-related substances in saliva may be altered under conditions of academic-related chronic stress, creating a favorable environment for the growth of *S*. *moorei* and its interaction with other bacteria species, such as *F*. *nucleatum*, resulting in an increase in VSCs production in the oral cavity. Another possibility is that stress-related substances might not affect bacterial growth or interaction, but upregulate the genes involved in the production of VSCs, as shown in previous work, where it was found that adrenaline and noradrenaline upregulated the expression of virulence and oxidative stress genes of *P*. *gingivalis* [18]. These genes may be related to β-galactosidases (deglycosylating enzymes) [46] or METase enzymes, which are responsible for CH_3_SH formation [48]. Future studies should be conducted in order to confirm the interactions between stress substances and bacterial VSCs production.

*T*. *forsythia* was found in very low relative proportion in both “Stressed” and “Not stressed” groups. However, this species showed moderate positive correlation with *S*. *moorei* and *F*. *nucleatum*, only in stressed subjects, suggesting that it might be related to them under stress conditions.

*T*. *forsythia* has been found in greater proportion in subjects with halitosis [11], but it is rarely found in healthy subjects [49]. To our knowledge, no other study has demonstrated any correlation among *S*. *moorei*, *F*. *nucleatum*, and *T*. *forsythia*, and additional studies are necessary to elucidate these relationships.

*S*. *moorei* was also moderately correlated with *A*. *odontolyticus* in both “Stressed” and “Not stressed” groups, coexisting with this bacterium in saliva samples of these volunteers. These correlations suggest that these bacteria might interact with each other, while stress was not able to stimulate this phenomenon and did not influence VSCs production.

Volunteers of the “Stressed” group showed increased oral emanation of (CH_3_)_2_S. The production of this gas has been reported to be associated with extra-oral halitosis [35], and its presence in the oral cavity could also be associated with its release from blood to saliva. The bacterial species evaluated here do not usually produce detectable levels of (CH_3_)_2_S, as reflected by the absence of correlation between the bacteria and (CH_3_)_2_S production. However, it is possible that other bacteria could be responsible for its production. *Pseudomonas aeruginosa* has been identified as a producer of this gas [50], but was not evaluated in the present study, because this bacterium is not associated with halitosis.

## Conclusions

Academic-related chronic stress conditions can stimulate the production of H_2_S in healthy males with no diagnosis of halitosis, by increasing levels of *S*. *moorei* in the presence of *F*. *nucleatum*. *T*. *forsythia* may also participate in this process. The findings indicate that these bacteria are important contributors to increased H_2_S emanations due to emotional changes in male subjects. The reason for increased (CH_3_)_2_S production in such individuals remains unclear.

## Supporting information

S1 Fig English MBI-SS questionnaire. English version of the Maslach Burnout Inventory Student Survey questionnaire.
(DOC)

S2 Fig Portuguese MBI-SS questionnaire. Portuguese version of the Maslach Burnout Inventory Student Survey questionnaire.
(DOC)

S1 File Clinical data. Clinical parameters obtained in the present study.
(XLSX)
