# Supplementary material for: Changes in salivary microbiota increase volatile sulfur compounds production in healthy male subjects with academic-related chronic stress
Source: PLoS One. 2017 Mar 20;12(3):e0173686. doi: 10.1371/journal.pone.0173686 (PMC5358872; doi:10.1371/journal.pone.0173686)
Supplement: S1 Fig — English version of the Maslach Burnout Inventory Student Survey questionnaire. (DOC) [file pone.0173686.s001.doc]

| **MBI-SS (Maslach Burnout Inventory- Student Survey)** | | | | | | | | | | | |
| --- | --- | --- | --- | --- | --- | --- | --- | --- | --- | --- | --- |
| **Name:_____________________________________________ Age:________ Date:____/____/____** | | | | | | | | | | | |
|  |  |  |  |  |  |  |  |  |  |  |  |
| **0 – NEVER 1 - ONCE A YEAR OR LESS 2 – ONCE A MONTH OR LESS 3 - SOMETIMES IN THE MONTH**  **4 – ONCE A WEEK 5 – SOMETIMES IN THE WEEK 6 - ALWAYS** | | | | | | | | | | | |

| **0** | | **1** | **2** | **3** | | **4** | **5** | **6** |  | | | | | | | | | |
| --- | --- | --- | --- | --- | --- | --- | --- | --- | --- | --- | --- | --- | --- | --- | --- | --- | --- | --- |
|  | I feel emotionally drained by my studies. | | | | | | | | | | |  |  |  |  |  |  |  |
|  | I feel used up at the end of a day at university. | | | | | | | | | | |  |  |  |  |  |  |  |
|  | I feel tired when I get up in the morning and I have to face another day at the university. | | | | | | | | | | |  |  |  |  |  |  |  |
|  | Studying or attending a class is really a strain for me. | | | | | | | | | | |  |  |  |  |  |  |  |
|  | I feel burned out from my studies. | | | | | | | | | | |  |  |  |  |  |  |  |
|  | I have become less interested in my studies since my enrollment at the university. | | | | | | | | | | |  |  |  |  |  |  |  |
|  | I have become less enthusiastic about my studies. | | | | | | | | | | |  |  |  |  |  |  |  |
|  | I have become more cynical about the potential usefulness of my studies. | | | | | | | | | | |  |  |  |  |  |  |  |
|  | I doubt the significance of my studies. | | | | | | | | | | |  |  |  |  |  |  |  |
|  | I can effectively solve the problems that arise in my studies. | | | | | | | | | | |  |  |  |  |  |  |  |
|  | I believe that I make an effective contribution to the classes that I attend. | | | | | | | | | | |  |  |  |  |  |  |  |
|  | In my opinion, I am a good student. | | | | | | | | | | |  |  |  |  |  |  |  |
|  | I feel stimulated when I achieve my study goals. | | | | | | | | | | |  |  |  |  |  |  |  |
|  | I have learned many interesting things during the course of my studies. | | | | | | | | | | |  |  |  |  |  |  |  |
|  | During class I feel confident that I am effective in getting things done. | | | | | | | | | | |  |  |  |  |  |  |  |
|  | | | | | | | | | | | **PARTIAL SCORE** |  |  |  |  |  |  |  |
| **TOTAL SCORE** | | | | |  | | | | |  | | | | | | | | |
