# Supplementary material for: Changes in salivary microbiota increase volatile sulfur compounds production in healthy male subjects with academic-related chronic stress
Source: PLoS One. 2017 Mar 20;12(3):e0173686. doi: 10.1371/journal.pone.0173686 (PMC5358872; doi:10.1371/journal.pone.0173686)
Supplement: S2 Fig — Portuguese version of the Maslach Burnout Inventory Student Survey questionnaire. (DOC) [file pone.0173686.s002.doc]

| **MBI-SS (Maslach Burnout Inventory- Student Survey)** | | | | | | | | | | | |
| --- | --- | --- | --- | --- | --- | --- | --- | --- | --- | --- | --- |
| **Nome:_____________________________________________ Idade:________ Data:____/____/____** | | | | | | | | | | | |
|  |  |  |  |  |  |  |  |  |  |  |  |
| **0 - NUNCA 1 - UMA VEZ AO ANO OU MENOS 2 – UMA VEZ AO MÊS OU MENOS 3 - ALGUMAS VEZES AO MÊS**  **4 - UMA VEZ POR SEMANA 5 - ALGUMAS VEZES POR SEMANA 6 - TODOS OS DIAS** | | | | | | | | | | | |

| **0** | | **1** | **2** | **3** | | **4** | **5** | **6** |  | | | | | | | | | |
| --- | --- | --- | --- | --- | --- | --- | --- | --- | --- | --- | --- | --- | --- | --- | --- | --- | --- | --- |
|  | Sinto-me emocionalmente esgotado pelos meus estudos | | | | | | | | | | |  |  |  |  |  |  |  |
|  | Eu questiono o sentido e a importância de meus estudos | | | | | | | | | | |  |  |  |  |  |  |  |
|  | Tenho aprendido muitas coisas interessantes no decorrer dos meus estudos | | | | | | | | | | |  |  |  |  |  |  |  |
|  | Sinto-me esgotado no fim de um dia em que tenho aula | | | | | | | | | | |  |  |  |  |  |  |  |
|  | Durante as aulas, sinto-me confiante: realizo as tarefas de forma eficaz | | | | | | | | | | |  |  |  |  |  |  |  |
|  | Sinto-me cansado quando me levanto para enfrentar outro dia de aula | | | | | | | | | | |  |  |  |  |  |  |  |
|  | Sinto-me estimulado quando concluo com êxito a minha meta de estudos | | | | | | | | | | |  |  |  |  |  |  |  |
|  | Estudar e freqüentar as aulas são, para mim, um grande esforço | | | | | | | | | | |  |  |  |  |  |  |  |
|  | Tenho tornado-me menos interessado nos estudos desde que entrei nesta universidade | | | | | | | | | | |  |  |  |  |  |  |  |
|  | Tenho tornado-me menos interessado nos meus estudos | | | | | | | | | | |  |  |  |  |  |  |  |
|  | Considero-me um bom estudante | | | | | | | | | | |  |  |  |  |  |  |  |
|  | Sinto-me consumido pelos meus estudos | | | | | | | | | | |  |  |  |  |  |  |  |
|  | Posso resolver os problemas que surgem nos meus estudos | | | | | | | | | | |  |  |  |  |  |  |  |
|  | Tenho estado mais descrente do meu potencial e da utilidade dos meus estudos | | | | | | | | | | |  |  |  |  |  |  |  |
|  | Acredito que eu seja eficaz na contribuição das aulas que freqüento | | | | | | | | | | |  |  |  |  |  |  |  |
|  | | | | | | | | | | | **TOTAL PARCIAL** |  |  |  |  |  |  |  |
| **TOTAL GERAL** | | | | |  | | | | |  | | | | | | | | |
